# Implementing comprehensive geriatric assessment in an academic hematologic outpatient setting: experiences from medical doctors and patients

**DOI:** 10.3389/fonc.2025.1570889

**Published:** 2025-08-13

**Authors:** Steven Pierre Hiltgen, Heike A. Bischoff-Ferrari, Michael Gagesch, Alexander Kaltenborn, Markus G. Manz, Max J. Rieger, Rahel Schwotzer, Sebastian M. Stolz, Thorsten Zenz, Wiebke Rösler

**Affiliations:** ^1^ Department of Medical Oncology and Hematology, University Hospital Zurich, Zurich, Switzerland; ^2^ Centre on Aging and Mobility, University of Zurich, Zurich, Switzerland; ^3^ Department of Geriatrics and Aging Research, University of Zurich, Zurich, Switzerland; ^4^ University Clinic for Aging Medicine, Stadtspital Zürich, Zurich, Switzerland; ^5^ KAMIQ Institute, Detern, Germany

**Keywords:** geriatric management, hematologic malignancies, implementation of geriatric assessment, patients’ perspective, geriatric hematology

## Abstract

**Introduction:**

The incidence of most cancers increases with age and cancer is a leading cause of morbidity and mortality in the older population. Older cancer patients frequently have additional co-morbidities and functional decline, which can substantially affect treatment outcomes. Major oncology societies recommend a screening for geriatric impairments in patients at risk (e.g. G8), followed by a comprehensive geriatric assessment (CGA). Nevertheless, CGA is often not established in routine care. We describe the implementation process of CGA in patients with hematological cancers at the University Hospital of Zurich. We evaluate its benefits, its perception by physicians and patients, and identify potential obstacles and solutions to allow integration into daily clinical practice.

**Materials and Methods:**

This retrospective, single-center observational study was conducted at the University Hospital of Zurich. Patients aged ≥65 years with hematological malignancies who underwent CGA within the last 5 years were included. Patients were referred for CGA based on physicians’ choice. All data were extracted from electronic medical records and later analyzed. Perception of the CGA by patients and physicians was assessed by a questionnaire.

**Results:**

46 patients who underwent CGA between April 2019 and July 2023 were included in this study. 89.1% showed at least one impaired domain in the CGA. For 98% of patients, one or more interventions were suggested. Low G8 scores were significantly associated with detected CGA-impairments (p<0.05). 70% of patients found the CGA and its resulting recommendations useful and reported benefiting from the process. 75% of the physicians rated the resulting CGA report as helpful for their clinical assessment.

**Conclusion:**

Our data support the use of a CGA in older patients with hematological cancers based on positive feedback on its implementation, from both patients and treating physicians. Our results emphasize the need for a dedicated geriatric assessment in an older cancer population as it contributes to a more comprehensive medical evaluation and potentially improves overall care and quality of life for patients.

## Introduction

Cancer is widely recognized as a disease of the older population (1). Incidence rates of most cancers increase significantly with age and overall, cancer ranks as the second leading cause of death among older adults (1, 2). Hematological neoplasms account for approximately 9% of all cancers and are the fourth most diagnosed cancer in both sexes (3). The median age at diagnosis for these malignancies is 67 years (4). Projections indicate that by 2040, the incidence of hematologic malignancies in older adults may increase by up to 40% compared to the current rate (5). Despite advancements in personalized cancer treatments, older patients with hematologic cancers in particular, often present with very specific challenges, including impairments in multiple organ functions, inadequate nutritional intake, and cognitive or functional decline (6). These factors contribute strongly to an increased vulnerability to treatment-related stressors in older adults, potentially affecting overall outcomes (7–9).

In clinical practice, the functional status of a cancer patient is commonly assessed by his/her medical history, physical status, and the Eastern Cooperative Oncology Group Performance Status (ECOG-PS). However, the predictive value of the ECOG score might not be sufficient to assess fitness of older patients (10, 11). To improve care and risk-stratification for this particular patient population, major professional organizations like the International Society for Geriatric Oncology (SIOG), the American Society of Clinical Oncology (ASCO), and the European Society for Medical Oncology (ESMO) recently recommended a structured geriatric screening, *e.g.* the G8 screening questionnaire (12–16). The G8 questionnaire consists of eight questions that assess specific factors, such as mobility, nutrition, medication, and cognitive function. It has been demonstrated to be an effective screening tool in identifying patients who benefit most from an in-depth geriatric evaluation (15, 17, 18). If the G8 screening is positive (≤14 points), it should be followed by a Comprehensive Geriatric Assessment (CGA), a multidimensional, interdisciplinary diagnostic tool used to assess underlying medical, pharmacological, nutritional, psychological, and functional challenges of older adults, designed to identify and address areas of impairment (19). A CGA allows identification of otherwise under-recognized geriatric problems affecting different aspects of health, often undetected by routine examination (*e.g.* risk of falls, malnutrition or cognitive impairment) (20).

Consequently, CGA-based interventions hold the potential to reduce treatment-related toxicity and hospitalizations rates, may improve treatment-completion and reduce functional decline in hematological malignancies and other types of cancer (11, 21–24).

Outside of clinical trials, CGA is not yet part of routine care in daily practice of medical oncology and hematology. Frequently mentioned, potential obstacles include time constraints, the lack of trained staff, low financial compensation, and the complexity of the implementation process. However, these concerns appear only partially valid since the relative costs to perform a CGA compare favorably to today’s costs for exploring tumor characteristics by (functional) imaging modalities or genomic testing (25). Moreover, an existing “know-do gap” and a tendency to rely on traditional evaluation and treatment paradigms (*e.g.* ECOG) appear to hinder the routine implementation of CGA (10, 20, 25).

In 2019, a geriatric evaluation including a CGA was implemented at the University Hospital of Zurich for patients aged 65 years and older diagnosed with hematologic malignancies.

The data reported here is the first detailed evaluation of its implementation process, its usage, and its perception by treating physicians and patients. In addition, we address the obstacles encountered and propose potential solutions to overcome them.

## Methods

This retrospective, single-center observational study was conducted at the University Hospital of Zürich (USZ). Eligible patients were 65 years and older with hematological malignancies who underwent CGA between April 2019 and July 2023. All patients have consented to participate via a general consent form in accordance with the specifications of the ethics application for this study.

The only exclusion criterion was the lack of general consent.

### Ethics approval

The local ethics committee granted ethical approval for the study (BASEC No. 2024-00422) in accordance with the Declaration of Helsinki.

### CGA

Eligible patients were referred for CGA at the discretion of the treating hemato-oncologist based on age and clinical impression. The CGA was conducted exclusively by board certified geriatricians at the University Hospital of Zürich (USZ) and included a set of standardized and validated geriatric assessment tests covering the following domains: cognitive function, physical capacity (mobility, strength and risk of falls), nutritional status, sensory function (vision/hearing), frailty, polypharmacy, potentially inadequate medication, quality of life (QoL), mental health, risk for delirium and multimorbidity, basic activities of daily living (BADL, Barthel-Index) and instrumental activities of daily Living (iADL, Functional Activities Questionnaire).

The BADL domains captured by the Barthel-Index (26) represent the most basic activities involved in everyday independent function (bathing, dressing, eating, toileting, transferring, grooming, walking, using stairs). The iADL captured by the Functional Activities Questionnaire (27) describe activities necessary for adaptation to the environment and emphasize community-based tasks such as shopping, cooking, transportation, and housekeeping.

The specific tests and instruments are summarized in (**Table 1**). Based on the CGA’s results, tailored interventions were recommended within to the following domains: physical therapy and exercise, nutritional recommendations/nutrition counseling, screening for osteoporosis, evaluation of cognitive deficits, medication adaptation, referral to psycho-oncology, home-care-implementation and advanced care planning (ACP). Findings and recommended CGA-based interventions were documented in a medical report and filed in the patients’ medical records.

**Table 1:** Comparison of CGA-components and instruments with the G8 screening tool.

Components	CGA	G8 Screening
	*Instruments*	*Domains*
Cognitive function	MMSE, MoCA	Memory impairment
Mobility and muscle strength	SPPB, Hand-grip strength history of falls	Independent mobility
Nutrition	MNA, BMI	BMI, Weight loss
Frailty	Frailty-Phenotype	Self-perceived health
Activities of daily living (BADL, iADL)	Barthel-Index, FAQ	-not covered
Sensory function (vision/hearing)	Jaeger-board, Whisper-Test	-not covered
Polypharmacy and risk of potentially inadequate medication	STOPP/START, Beers List	Number of long-term medications
Quality of life and mental health	EuroQoL	-not covered
Multimorbidity	Sanghas-Questionnaire	-not covered
Age		by group (< 80 yrs, 80–85 yrs, > 85 yrs)

CGA, comprehensive geriatric assessment; MMSE, Minimal-Mental-State-Examination; MoCA, Montreal Cognitive Assessment; SPPB, short physical performance battery; MNA, Mini Nutritional Assessment; BMI, Bodymass-Index; BADL, Basic Activities of daily living; iADL, instrumental activities of daily living; FAQ, Functional Activities Questionnaire.

### Questionnaire for patients

All included patients were invited to complete anonymously a questionnaire on a separate encounter following the CGA. The questionnaire contained six statements, rated on a Likert scale from 1 (not at all) to 5 (very accurate), and patients had the opportunity to comment on their ratings. The statements were as follows:

- The appointment at the geriatric department was found to be useful.
- The recommendations given, e.g. on nutrition or physiotherapy were useful.
- I was able to implement the addressed recommendations well.
- Overall, I have the impression that I benefited from this appointment.
- I would have appreciated a second appointment at the geriatric department (e.g. after completing therapy).
- In principle, I believe that this type of service is useful.

### Questionnaire for physicians

Treating physicians from the Department of Medical Oncology and Hematology were invited to participate anonymously in an online survey evaluating their awareness and utilization of CGA as well as their opinion on various aspects and individual components of the CGA. In addition, information on their demographics and education was collected. The survey was conducted on the Survio online platform (see full questionnaire in the **Supplementary Material**).

### Data collection

Data from all patients were extracted from their electronic medical records and later analyzed and interpreted by all authors. Collected data included demographics (sex, date of birth, date and cause of death [if applicable]), diagnosis, date of diagnosis, ECOG, administered anti-cancer therapy (including precise therapy-type and duration), date of CGA, CGA results and recommendations, disease outcome and recurrence, development of weight and serum albumin (measured at date of diagnosis, therapy-initiation, therapy-ending and last visit, data were accepted within a three-month time frame prior to or subsequent to the event in question).

### Statistical analysis

Statistical analyses were performed with IBM SPSS version 29 and the MedCalc v22 statistics software (28). The Kolmogorov-Smirnov test was used to assess the distribution of continuous data. In normally distributed data, results were presented by mean and standard deviation (SD); if a non-parametric distribution was detected, the data were presented by median and range. Inferential statistics were performed to identify relevant associations between the CGA and various outcome parameters.

For the comparison of non-parametrically distributed data, the Mann-Whitney-U test was applied. In case of parametric distribution, the data were compared with the Student’s two-sided t-Test. Categorical variables were analyzed with the Pearson’s Chi-squared test. A p-value <0.05 was defined as statistically significant.

## Results

### Patients’ characteristics and diagnoses

Between April 2019 and July 2023, 46 patients were referred for CGA. Median age was 75.5 years (62 to 88 years) and 69.6% (N=32) were male. The average duration of the follow-up from CGA to the last known contact was 579 days. 45.7% (N=21) were diagnosed with aggressive B-cell lymphoma, 21.7% (N=10) with indolent B-cell lymphoma, 17.4% (N=8) with multiple myeloma, 10.9% (N=5) with myelodysplastic syndrome (MDS)/acute myeloid leukemia (AML) and 4.3% (N=2) with Hodgkin’s lymphoma (HL). Patients’ characteristics are provided in detail in **Supplementary Tables 1, 2** **(Supplementary Material)**.

### CGA findings and recommendations

**Figure 1:**
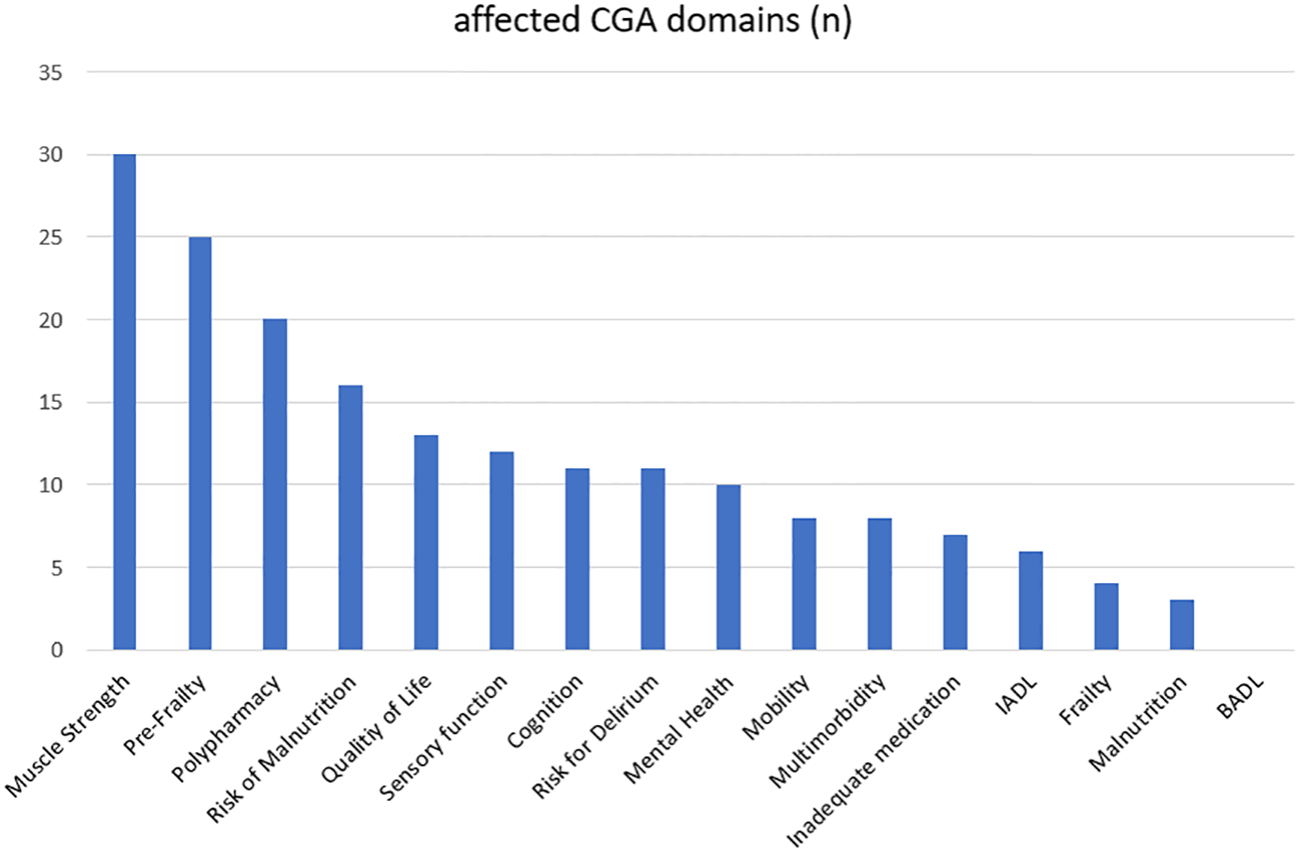
Affected CGA domains, n: absolute numbers.

**Figure 2:**
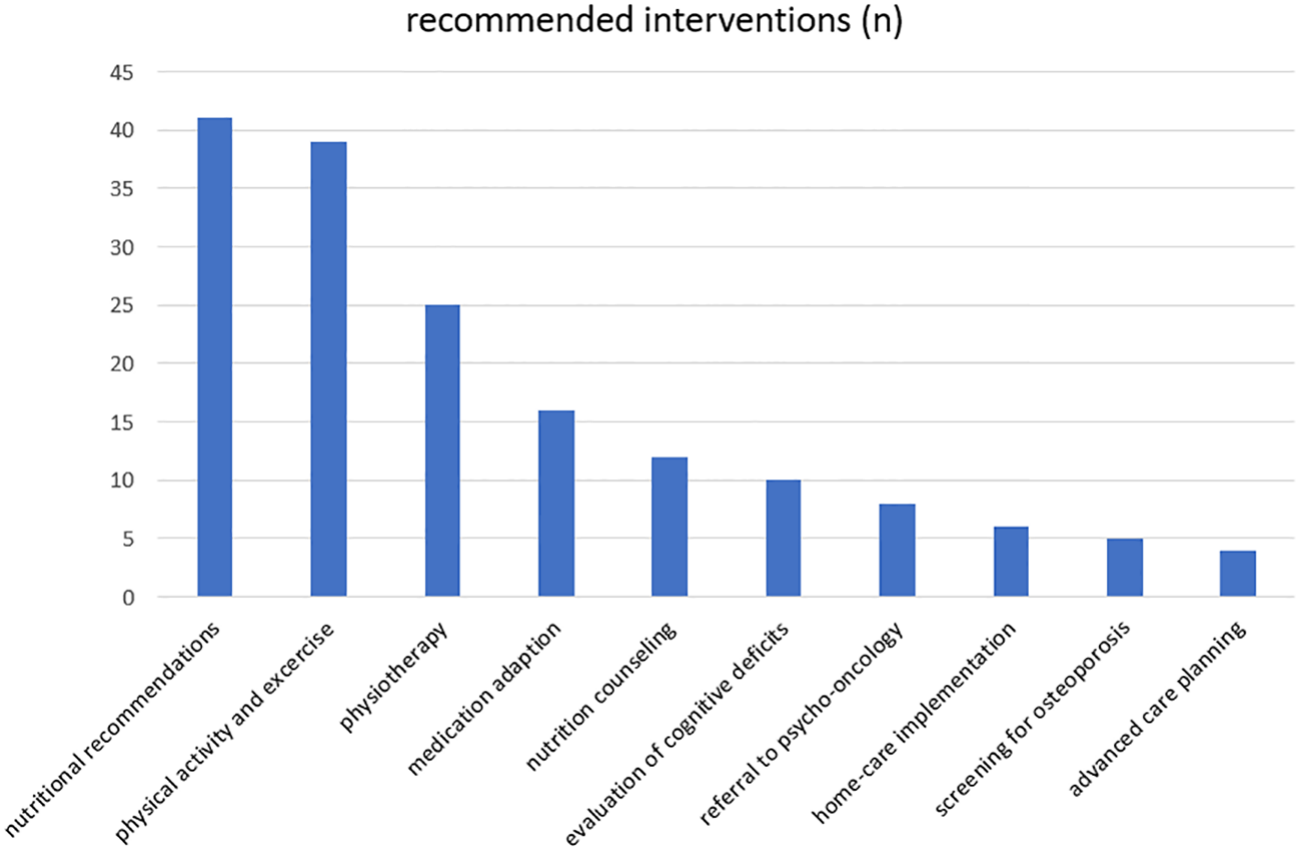
Recommended interventions, n: absolute numbers.

Time from first diagnosis to the CGA varied between 7 and 7,726 days, with a median of 224 days. The median number of affected CGA domains was 4 (range: 0-10). Among the 46 patients, 41 (89.1%) showed at least one impaired domain in the CGA, and 24 patients (52.2%) showed at least 4 affected CGA domains. The most affected domains were muscle strength (N=30, 65.2%), pre-frailty (N=25, 54.3%) and polypharmacy (N=20, 43.5%); detailed information is shown in the **Figure 1**. In 45 patients (97.8%) one or more interventions were recommended. The most frequently interventions were nutritional recommendations (N=41, 89.1%) and physical activity/exercise (N=39, 84.8%) while physical therapy was recommended in 54.3% (N=25), adaption of medication in 34.8% (N=16) and nutritional counseling in 26.1% (N=12) of cases. Detailed information is shown in the **Figure 2**.

### ECOG/G8-screening

Most patients with documented ECOG status (N=27, 58.7%) showed a PS of 0 (N=11, 40.7%) or 1 (N=11, 40.7%).

Only a small number of patients showed a PS of 2 (N=3, 11.1%) and PS of 3 (N=2, 7.4%). There was no significant correlation between ECOG-PS and detected impairments in CGA. G8-Score was available in 91.3% (N=42) of cases and 64.3% (N=27) of those patients had an abnormal low G8-Score ≤14 points. G8-Scores and pathological CGA-outcome (defined as results deviating from predefined validated limits for the specific test of CGA) were correlated (p = 0.011).

### Treatment outcomes and complications

At the time of the CGA, most patients (N=25, 54.3%) were undergoing intensive treatment (defined as treatment that uses anti-cancer drugs given at high doses or over several months intended to cure or induce a remission), reduced/palliative treatment (defined as treatment given to help relieve the symptoms, N=17, 37.0%), or watch & wait (N=4, 8.7%). At the time the CGA was performed, most patients receiving therapy were either undergoing their first line of treatment (N=25, 59.5%) or were already on their second (N=5, 11.9%), third, or later lines of therapy (N=14, 33.3%). Some patients underwent CGA after end of treatment (N=4, 9.5%).

A total of 26 patients (61.9%) achieved complete remission following the treatment received at the time of CGA, while 16.6% (N= 7) experienced a relapse or progressive disease, 14.3% (N=6) showed partial response and 7.1% (N=3) stable disease during the observed time-period (April 2019 to February 2024). There was no significant association between CGA and either the treatment choice or the remission rate.

A total of 33 patients (78.6%) had treatment associated complications, including infections (N=25, 59.5%), unplanned hospitalizations (N=21, 50%) or other complications (N=29, 69.0%). Abnormal findings in individual CGA domains were not correlated with complications or toxicity of treatment.

### Results from the patients’ questionnaire

37 out of 46 patients could be contacted for feedback. Of those, 19 patients answered the questionnaire. Results are shown in **Table 2**.

**Table 2:** Patients’ questionnaire, Likert scale from 1 point: not at all to 5: very accurate.

Question	Average rating (1 to 5)	Ratings ≥ 4 points
The appointment at the geriatric department was found to be useful.	3.94	68.4% (N: 13)
The recommendations given e.g. on nutrition or physiotherapy were useful.	4.36	73.7% (N: 14)
I was able to implement the addressed recommendations quite well.	3.64	63.2% (N: 12)
In total, I have the impression that I benefited from this appointment.	3.88	73.7% (N: 14)
I would have appreciated a second appointment at the geriatric department (e.g. after therapy-completion)	2.18	21.1% (N: 4)
In principle, I believe that this type of service is useful	4.18	68.4% (N: 13)

### Results from the physician’s questionnaire

45 (67.2%) of 67 contacted physicians completed the questionnaire (see full questionnaire in the **Supplementary Material**). Of those, 51.1% (N=23) identified as male, 44.4% (N=20) as female, and 4.4% (N=2) as diverse. Overall, 40% (N=18) were resident doctors, 28.9% (N=13) senior physicians with less than 10 years of work experience, and 31.1% (N=14) senior physicians with more than 10 years of work experience. Altogether, 68.9% (N=31) were working primarily in hematological oncology, and 31.1% (N=14) in medical oncology. Overall, only 11.1% (N=5) reported to have attended lectures during their studies specifically regarding geriatric oncology/hematology, while 88.9% (N=40) did not. 48.9% (N=22) reported they never had the chance to, since such lectures were never available at their medical school.

To assess the functional status, all physicians (N=45, 100%) relied on the patient’s medical history and clinical examination. In addition, 91.1% (N=41) used the ECOG Performance Status, 55.6% (N=25) used the Karnofsky Index, and 15.6% (N=7) also applied the G8 screening tool. For the evaluation of social support, all participating physicians again referred to the medical history (N=45, 100%), followed by information from relatives and caregivers (N=37, 82.2%). Only 11.1% (N=5) used data provided by the Comprehensive Geriatric Assessment (CGA).

The most frequently reported sources of information to evaluate the cognitive function, were the medical history and clinical examination (N=44, 97.8%), followed by information provided by relatives or caretakers (N=29, 64.4%), and available doctor’s reports (N= 3, 6,7%). 24.4% (N=11) of physicians used CGA. MMSE (N=12, 26.7%) and MoCA (N=5, 11.1%) were used infrequently.

A total of 24 physicians (53,3%) reported to had experience CGA in their daily work at the University Hospital, whilst 46.7% (N=21) had never used it before. The most common reason for non-referrals were unfamiliarity with the program (N=11, 52.4%), patients` wish (N=5, 23.8%) and unawareness of the benefits of CGA (N=3, 14.3%).

When CGA was conducted, 79% (N=19) rated the CGA-report helpful, mostly in the following domains: frailty assessment (3.9), cognition (3.8), nutritional status/optimization (3.7), physical function (3.7) and falls/risk of falls (3.6) (**Table 3**).

**Table 3:** Average-ratings on CGA domains by physicians, Likert scale from 1 point: not at all to 5: very accurate.

CGA domains	Average-Rating (0-5)
Frailty assessment	3.9
Cognition	3.8
Nutritional status/optimization	3.7
Physical function	3.7
Falls/risk of falls	3.6
Integration/utilization of additional resources (e.g. nutritional counseling or physiotherapy)	3.3
Management of non-oncological comorbidities and polypharmacy.	3.2
Pain management	3.0
Oncological treatment decision	2.5

## Discussion

In our single-center, retrospective observational study we describe the implementation of a CGA-based program in older patients with hematological malignancies. In our cohort, G8-scores correlated significantly with pathological CGA-outcomes. A G8 ≤14 was significantly correlated with affected CGA domains. This highlights the value of performing a CGA, as having at least one impaired CGA domain correlates with low G8 scores. While limited time, financial support and low awareness were among the main hurdles for CGA’s establishment, the generally positive *post-hoc* perception of the CGA and its proposed interventions by both patients and physicians demonstrate its benefits in clinical practice.

Our results are important because they provide insight into the feasibility of establishing a CGA program with CGA-driven interventions for older patients with hematologic cancers. They may potentially provide rational for a broader implementation and further development of the CGA-program. Especially the integration of a geriatric-oncological consultation into routine clinical practice, as well as the inclusion of geriatric-oncological aspects in medical training should be key-aspects in future developments. Furthermore, the recently introduced Practical Geriatric Assessment (PGA), which has been endorsed by ASCO experts, offers new possibilities for the implementation of an assessment tool in everyday practice (29).

With a median age of 75.5 years and a high heterogeneity in overall health status, our patients represent a group who is often under-represented in cancer clinical studies but mirrors the day-to-day experience and challenges in the clinical setting (30–32). CGA identified a considerable number of previously undetected functional impairments, which led to several CGA-based recommendations. Approximately 90% of patients showed at least one impaired CGA domain with 98% receiving at least one recommendation.

This observation aligns with existing data from clinical trials such as the GERICO trial or the GAP70+ study, both of which demonstrate a high prevalence of geriatric impairments in this population (21, 23). This underlines the relevance of CGA, as these impairments would have been likely missed and not addressed with supportive interventions in traditional management. In addition, ECOG is not sufficient to characterize older patients, as it was not correlated with the number of detected deficits in the different CGA domains nor need for interventions. This is in line with other publications and further underlines the need for a better approach for older cancer patients (33, 34).

According to our questionnaire for patients and physicians, CGA was rated as being a positive and useful intervention, aligning with findings from other trials that reported benefits such as improved decision-making (24), improved QoL (21, 22), increased likelihood of having end-of-life goals-of-care discussions (35) and improved patient-centered communication about aging-related concerns (36). Aside from our analysis, these results were primarily evaluated from the physicians’ perspective. Our results also highlight that, for older patients, minimizing the risk of toxicities and maximizing the function and quality of life are among the most important aspects of care (23, 36). Based on the physician’s questionnaire, geriatric oncology and hematology seem to be underrepresented in many medical curricula. This may contribute to the missing knowledge and low awareness of the potential role and benefits of CGA, which should be further addressed in the future.

Implementing a CGA before the start of anti-cancer treatment appears challenging. In our patient group, CGA was performed before treatment start in only 39.1% of cases (N: 18). There is accumulating evidence that CGA before treatment initiation can significantly impact treatment-planning including potential dose reduction based on CGA-findings (25). Patients with aggressive lymphoma or acute leukemia are often considered to benefit from immediate systemic treatment at diagnosis (37, 38). In contrast to solid tumors, a curative treatment of hematological malignancies mostly requires systemic therapies. These aspects make a timely implementation even more challenging.

Nevertheless, the time and costs required for CGA are just a fraction of the additional expenses associated with potential toxicity events and complications (25). Addressing the logistical and financial challenges appears crucial to support the CGA implementation process.

Our study’s limitations include its single-center design with a small sample size, which was further limited by the COVID-19 pandemic leading to slow recruitment. However, this reflected the real-world struggles in hematology departments in many parts of the world. Comparative data from our department in 2023 indicate that approximately 75 patients are eligible for CGA annually. Based on the reported data, we have now consistently implemented G8 screening for all patients >70 years (or >65 years with comorbidities), in line with current recommendations (31). In addition, the introduction of a clear process with screening tools leads to a more informed and reliable referral of patients, based not only on the physician’s choice but on a more objective approach. This, too, should reduce the bias we had during the implementation process. Screening all patients in the outpatients setting at first visit contributes to the issue of high heterogeneity in the timepoint of CGA. This heterogeneity in our data is mainly based on trying to gain first experience with the process and the idea, and not on withholding patients from CGA only because they had already started treatment.

Our new approach led to already 20 patients undergoing CGA within a four-month period since June 2024. The CGA includes all of the above named measurements as well as QoL aspects (measured by EORTC-QLQ). Also, the absence of a control group and the cohort’s heterogeneity in terms of diagnosis and therapies limit the generalizability of the results. Furthermore, there was no control or follow-up on the CGA recommendations, and implementation of recommendations was done based on the choice of the treating physicians. In the future, we will re-evaluate patients 3 months after the first CGA to evaluate the recommendations and their effects, which will hopefully lead to more meaningful results.

## Conclusion

Our data support the benefits of a CGA in older cancer patients by adding relevant information regarding functional status and overall patient care. CGA provides a more comprehensive evaluation of the medical status of the patients and offers an opportunity to clarify the patient’s priorities. Overall, it is perceived as helpful by both patients and physicians. Therefore, earlier and closer collaboration between oncologists/hematologists and geriatricians appears to improve routine care of older patients. Integration of a CGA into the routine hematologic/oncologic practice may lower the barrier to referring patients. Higher awareness about geriatric oncology should be a priority in both early medical training and ongoing professional education.

## Data Availability

The raw data supporting the conclusions of this article will be made available by the authors, without undue reservation.
