# Supplementary material for: Implementing comprehensive geriatric assessment in an academic hematologic outpatient setting: experiences from medical doctors and patients
Source: Front Oncol. 2025 Aug 13;15:1570889. doi: 10.3389/fonc.2025.1570889 (PMC12380937; doi:10.3389/fonc.2025.1570889)
Supplement: Supplementary file 1 [file DataSheet1.pdf]

Supplementary Material:

1) **Questionnaire for physicians (translated from German)**

1. I am familiar with G8 and Comprehensive Geriatric Assessment (CGA): Yes/No/partly

2. I am aware that the assessment of the G8 before the start of oncological therapy is part of important oncological guidelines (ASCO, NCCN, SIOG)*: Yes/No

3. I assess the general condition of older cancer patients by using (multiple answers possible)*:
   - ECOG
   - medical history and clinical examination
   - KPS
   - geriatric screening tools (such as G8)
   - history of falls
   - 10 m walking test
   - something different (please explain)

4. I assess the required social support for older cancer patients by (multiple answers possible)*
   - medical history and clinical examination
   - geriatric assessment
   - relatives/care givers
   - I don't do this myself, but someone else from the team (nursing, social services)
   - not at all
   - something different (please explain)

5. I assess the cognition of older cancer patients using (multiple answers possible)*
   - medical history and clinical examination
   - MMSE (Minimal Mental Status Examination)
   - MOCA (Montreal Cognitive Assessment)
   - medical reports
   - I refer the patient to the CGA
   - relatives
   - something different (please explain)

6. I have already referred patients to the CGA*: Yes/No

7. If the previous question 6 was answered with 'No': Why not?
   - I was not aware of the service
   - benefits are not clear to me
   - patient refused
   - no time before/during therapy
   - something different (please explain)

8. If the answer to question 6 was 'Yes', did you find the CGA report helpful?: Yes/No

9. If question 6 was answered with 'Yes': Were recommendations from the CGA useful?*: Yes/No

10. If there were recommendations after the CGA: Were they implemented?*: Yes/No

11. If the recommendations have not been implemented, why not?
    - patient refused
    - recommendations were not prescribed/responsibilities were not clear
    - have not seen any benefit in this
    - something different (please explain)

12. If there is experience with the CGA: On a scale of 1 (not very helpful) to 5 (very helpful), how would you rate the CGA in the following areas:
    - assessment of depression
    - analgetic therapy
    - nutritional status/optimization
    - falls/risk of falls
    - cognition
    - physical function
    - frailty
    - management of non-oncological co-morbidities and polypharmacy
    - involvement/utilization of additional resources (e.g. nutritional counselling/physiotherapy)
    - therapy decisions (for malignant disease)

13. The G8 as a screening test should be recorded and documented by:*
    - doctors
    - nurses
    - non-medical staff
    - other options (please explain)
    -

14. During my university studies, I attended lectures/courses on the subject of geriatric oncology/haematology?*: Yes/No /not offered

15. I work mainly at:*
    - Hematology
    - Oncology

Biographic data: I am: Fellow
Attending < 10 yrs of professional experience
Attending > 10 yrs of professional experience

Male/female/divers

*: mandatory questions

**Patient Characteristics**	N (%)
**Sex**	
Male	32 (69.6%)
Female	14 (30.4%)
**Age at CGA (years)**	
Median	75.5 (62-88)
**ECOG performance status**	
0	11 (23.9%)
1	11 (23.9%)
2	3 (6.5%)
3	2 (4.3%)
Not available	19 (41.3%)
**G8 Score**	
> 14 Points	15 (32.6%)
≤ 14 points	27 (58.7%)
Not available	4 (8.7%)
Timepoint of CGA	
Before start of treatment	18 (39.1%)
After start of treatment	26 (56.5%)
No treatment	2 (4.3%)

supplementary Tab. 1.: Patient Characteristics

**Cancer type**	
Aggressive B-cell lymphoma	21 (45.7%)
Indolent B-cell lymphoma	10 (21.7%)
Multiple Myeloma	8 (17.4%)
MDS/AML	5 (10.9%)
HL	2 (4.3%)
**Number of Treatment-line(s)**	
0	2 (4.3%)
1	25 (54.3%)
2	5 (10.9%)
>2	14 (30.4%)
**Treatment intensity**	
intensive	25 (54.3%)
reduced/palliative	17 (37.0%)
watch & wait	4 (8.7%)
**Treatment-Outcome**[3]	
Complete remission	26 (61.9%)
Partial remission	6 (14.3%)
Stable disease	3 (7.1%)
Progressive disease/Relapse	7(16.6%)

supplementary Tab. 2 Patients' diagnoses and treatment details

*= percentages refer to patients undergoing treatment (42/46)
